# Lipoma in dogs under primary veterinary care in the UK: prevalence and breed associations

**DOI:** 10.1186/s40575-018-0065-9

**Published:** 2018-09-27

**Authors:** Dan G. O’Neill, Caroline H. Corah, David B. Church, Dave C. Brodbelt, Lynda Rutherford

**Affiliations:** 1 Production and Population Health, The Royal Veterinary College, Hawkshead Lane, North Mymms, Hatfield, Herts AL9 7TA UK; 2 Clinical Sciences and Services, The Royal Veterinary College, Hawkshead Lane, North Mymms, Hatfield, Herts AL9 7TA UK

**Keywords:** General practice, First opinion, VetCompass, Fatty mass, Lipoma

## Abstract

**Background:**

Lipomas are masses of mesenchymal origin, comprising of adipocytes, and are often clinically unremarkable but can be alarming to owners. Although lipomas are reportedly common in dogs, no studies have specifically investigated risk factors associated with their occurrence. This study was a large-scale retrospective analysis of electronic patient records of dogs attending practices participating in VetCompass™. Univariable and multivariable logistic regression methods were used to evaluate associations between risk factors and primary-care veterinary diagnosis of lipoma.

**Results:**

From 384,284 dogs under veterinary care during 2013 at 215 primary practice clinics in the UK, there were 2765 lipoma cases identified giving a one-year prevalence of 1.94% (95% CI: 1.87–2.01). Breeds with the highest lipoma prevalence included Weimaraner (7.84%, 95% CI 6.46–9.40), Dobermann Pinscher (6.96%, 95% CI 5.67–8.44), German Pointer (5.23%, 95% CI 3.93–6.80), Springer Spaniel (5.19%, 95% CI 4.76–5.66), and Labrador Retriever (5.15%, 95% CI 4.90–5.41). Dogs with an adult bodyweight equal or higher than their breed/sex mean had 1.96 (95% CI 1.81–2.14, *P* <  0.001) times the odds of lipoma compared with dogs that weighed below their breed/sex mean. The odds of lipoma increased as adult bodyweight increased. Increased age was strongly associated with increasing odds of lipoma. Compared with dogs aged 3.0 to < 6.0 years, dogs aged 9.0 - < 12.0 years had 17.52 times the odds (95% CI 14.71–20.85, *P* <  0.001) of lipoma. Neutered males (OR: 1.99, 95% CI 1.69–2.36, *P* <  0.001) and neutered females (OR: 1.62, 95% CI 1.37–1.91, *P* <  0.001) had higher odds than entire females. Insured dogs had 1.78 (95% CI 1.53–2.07, *P* <  0.001) times the odds of lipoma compared with uninsured dogs.

**Conclusions:**

Lipomas appear to be a relatively common diagnosis in primary-care practice. Certain breeds were identified with remarkably high lipoma prevalence, highlighting the risk that owners should be prepared for. Lipoma predisposition of larger bodyweight individuals within breed/sex suggests that being overweight or obese may be a predisposing factor but would need further work to confirm.

## Plain English summary

Lipomas are fatty masses that may not cause dogs substantial direct problems but can often cause severe anxiety to owners when there are multiple or very large masses. These masses were the most common disorder reported in dogs in a large UK survey of pedigree dog owners. However, despite this, there has been very little information published on which breeds are most affected even though this information would be very relevant to owners. First opinion veterinary health records are now recognised as a valuable source of information to learn about the diseases that are diagnosed in dogs by veterinarians. This study aimed to use the VetCompass™ database of veterinary health records to explore how common lipomas were in the UK and to identify which breeds were most affected.

The study included 384,284 dogs under veterinary care at 215 clinics in the UK during 2013. Overall, 1.94% of the dogs were diagnosed with lipoma. The breeds that were most commonly diagnosed were Weimaraner (7.84%), Dobermann Pinscher (6.96%), German Pointer (5.23%), Springer Spaniel (5.19%), and Labrador Retriever (5.15%). Eleven breeds including the Yorkshire Terrier, Lhasa Apso, German Shepherd Dog and Shih-tzu had reduced risk of lipoma. As dogs aged, they were much more likely to have lipoma. Neutered males and neutered females had higher risk than entire females. Insured dogs were almost twice as likely to be diagnosed compared with uninsured dogs. Purebred dogs and heavier individuals were also more likely to be diagnosed.

This large population study is the first study to focus primarily on lipoma occurrence in the dogs in the UK. Lipoma has been confirmed as a common diagnosis in dogs. The strong breed associations shown for both lipoma predisposition and protection can assist with breed health reforms as well as preparing owners of predisposed breeds for the probability of this alarming clinical presentation.

## Background

Lipomas are masses of mesenchymal origin, comprising of adipocytes, and are often clinically unremarkable [1–3]. Individual case reports or case series have reported on intra-cavity lipomas and lipomas adjacent to neural structures, which have caused clinical signs due to organ compression [2, 4–6]. Although subcutaneous lipomas are often clinically asymptomatic [7], they can cause deleterious consequences for the patients and anxiety for the owners if they become sizable or interfere with locomotion [1, 7, 8].

Lipomas of the dermis and subcutaneous tissues are reported to be common in older dogs [1] and were the most common disorder of purebred dogs in the UK in an owner-reported survey [9]. Lipomas were the 12th most commonly reported disorder in dogs in the south of England with a prevalence of 3.5% reported from a sample of 3884 dogs under primary veterinary care [10]. Fatty tumours were reported as the most common tumour diagnosed by cytology in Dutch Golden Retrievers [11]. Lipomas were the most common benign tumour (24%) identified in the Danish Cancer Registry [12] and were the second most common tumor recorded in insured dogs in the UK with an incidence rate of 337 per 100,000 dogs per year [13].

Despite the evidence showing relatively frequent occurrence in dogs, there is very little published evidence on risk factors for lipomas. Advancing age, overweight dogs and females have been suggested as having increased risk [14]. Dobermann Pinscher and Labrador Retriever have also been reported as predisposed breeds [15]. However, little other information on risk factors for lipoma have been published.

Primary-care veterinary clinical data are now recognised as a valuable research resource that benefit from contemporaneous recording of medical records at the time of the clinical event, and from the recording of cohort data over time and at a veterinary level of precision [16, 17]. Such data have been validated for research purposes by several previous reports on diverse conditions in dogs including road traffic accidents [18], appendicular osteoarthritis [19], dystocia [20], urinary incontinence [21] and corneal ulcerative disease [22]. This study aimed to fill the information gap on the epidemiology of lipoma by estimating the prevalence of lipoma, and evaluating demographic risk factors for lipoma in the dog population under primary-care veterinary care in the UK. Based on the prior but scant information in the literature, it was hypothesized that purebred dogs, older dogs and heavier dogs would have higher odds of lipoma than crossbred, younger and lighter dogs respectively.

## Methods

The VetCompass™ Programme collates de-identified electronic patient record (EPR) data from primary-care veterinary practices in the UK for epidemiological research [10]. Collaborating practices can record summary diagnosis terms from an embedded VeNom Code [23] list during episodes of care. VetCompass™ collects information fields that include species, breed, date of birth, sex, neuter status, insurance status and bodyweight, and clinical information from free-form text clinical notes and summary diagnosis terms (VeNom codes), plus treatment and deceased status with relevant dates. The EPR data were extracted from practice management systems using integrated clinical queries and uploaded to a secure VetCompass™ structured query language database [24].

A cross-sectional analysis using cohort clinical data of dogs attending VetCompass™ practices was used to estimate the prevalence and risk factors for lipoma [25]. The sampling frame for the current study included dogs under veterinary care within the VetCompass™ database for a one-year period from January 1st 2013 to December 31st 2013. Dogs ‘under veterinary care’ were defined as any dog with either at least one EPR recorded from January 1st to December 31st 2013 or, alternatively, at least one EPR both before and after 2013. Sample size calculations estimated that a cross-sectional analysis would require a sample size of 108,980 dogs to provide a prevalence estimate for a disorder expected to occur in 1.0% of overall population with a 0.05% confidence limit assuming a UK population size of 8,000,000 dogs [26, 27]. Ethical approval was granted by the RVC Ethics and Welfare Committee (reference number 2016/U37).

Case inclusion criteria required that a final diagnosis of lipoma (or synonym) was recorded in the EPR for a mass at any body location that was present during the 2013 study period. The clinical decision-making used for diagnosis of lipoma was at the discretion of the attending veterinary surgeon. Case-finding involved initial screening of all EPRs for candidate lipoma cases by searching the clinical free-text field using search terms including lipo*, fat* + mass*, fat* + lump*, fat* + FNA*, fat* + biop*, and the VeNom term field using the search term *lipoma*. The candidate cases were randomly ordered by the Microsoft Excel *RAND* function (Microsoft Office Excel 2007, Microsoft Corp.) and the clinical notes of a random subset of candidate cases were manually reviewed in detail to evaluate for case inclusion. All dogs that were not identified as candidate lipoma cases during the initial screening were included as non-cases and all dogs that met the inclusion criteria for lipoma were grouped as *lipoma cases* in the risk factor analysis.

A *purebred* variable categorised all dogs of recognisable breeds as ‘purebred’ and the remaining dogs as ‘crossbred’ [28]. A *breed* variable included individual breeds represented by over 4000 dogs in the overall study or with ≥15 lipoma cases, a grouped category of all remaining purebreds and a general grouping of crossbred dogs. This approach was taken to facilitate statistical power for the individual breed analyses [29]. A *Kennel Club breed group* variable classified breeds recognised by the UK Kennel Club into their relevant breed groups (Gundog, Hound, Pastoral, Terrier, Toy, Utility and Working) and all remaining types were classified as non-Kennel Club recognised [30]. *Sex* (female, male, unrecorded) and *neuter* (neutered, entire, unrecorded) variables described the status recorded at the final EPR. Sex and neuter status were also combined into a single variable that reported the results across seven permutational categories. An *insurance* variable described whether a dog was insured at any point during the study period. Age (years) was calculated for all dogs at the final date of the study period (December 31st, 2013). An *age* variable categorised age (years) into six groups (< 3.0, 3.0 - < 6.0, 6.0 - < 9.0, 9.0 - < 12.0, ≥ 12.0, unrecorded). Adult bodyweight described the maximum bodyweight (kg) recorded for dogs > 18 months old. An *adult bodyweight* variable categorised adult bodyweight (kg) into six groups (< 10.0, 10.0–19.9, 20.0–29.9, 30.0–39.9, ≥ 40.0, not available). A *bodyweight relative to breed mean* variable characterised the adult bodyweight of individual dogs as either below or equal/above the mean adult bodyweight for their breed and sex within the overall study population. This variable allowed the effect of adult bodyweight to be assessed *within* each breed/sex combination.

Following data checking and cleaning in Excel (Microsoft Office Excel 2013, Microsoft Corp.), analyses were conducted using Stata Version 13 (Stata Corporation). The one-year period prevalence with 95% confidence intervals (CI) described the probability of evidence in the clinical records that confirmed the presence of lipoma at any time during the one-year 2013 study period. Because the sampling design involved manual verification of a subset of the candidate cases, the predicted case count was calculated using the Stata *survey* function that weighted the verified case numbers by the inverse of the proportion of candidate cases manually confirmed [24]. The CI estimates were derived from standard errors, based on approximation to the normal distribution [31]. Descriptive statistics characterised the risk factors separately for the case and non-case dogs.

Binary logistic regression modelling was used to evaluate univariable associations between risk factors (*purebred, breed, Kennel Club breed group, adult bodyweight, bodyweight relative to breed/sex mean, age, sex, neuter, sex-neuter* and *insurance*) and diagnosis of lipoma during 2013. Because breed was a factor of primary interest for the study, *purebred,* and *Kennel Club breed group* (variables that are highly collinear with breed) and *adult bodyweight* (a defining characteristic of individual breeds) were excluded from the initial breed multivariable modelling. Instead, each of these variables individually replaced the *breed* variable in the main final model in order to evaluate their effects after taking account of the other variables. Risk factors with liberal associations in univariable modelling (*P* <  0.2) were taken forward for multivariable evaluation. Model development used manual backwards stepwise elimination. Clinic attended was evaluated as a random effect and pair-wise interaction effects were evaluated for the final model variables [32]. The area under the ROC curve and the Hosmer-Lemeshow test were used to evaluate the quality of the model fit and discrimination (non-random effect model) [32, 33]. Statistical significance was set at *P* <  0.05.

## Results

**Fig. 1:**
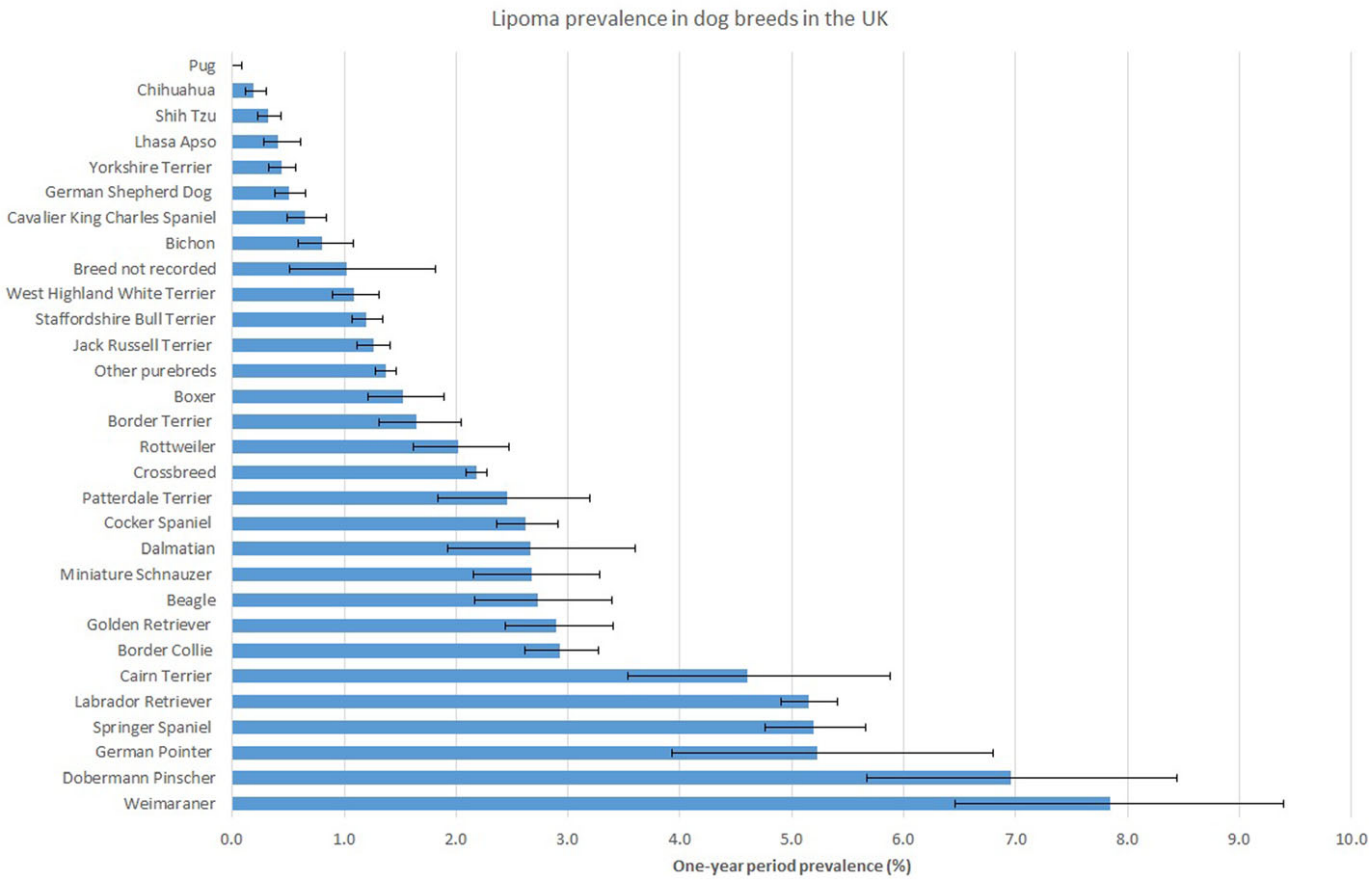
One-year (2013) period prevalence of lipoma diagnosis in commonly affected dog breeds attending primary-care veterinary practices in the VetCompass™ Programme in the UK. The error bars show the 95% confidence interval

The denominator population comprised 384,284 dogs under veterinary care at 215 clinics in the UK during 2013. Of 8437 candidate cases identified, 3504 (36.5%) were manually checked to confirm 2765 lipoma cases from this sample. After accounting for the effects of the subsampling protocol, the estimated one-year period prevalence for lipoma diagnosis in dogs overall was 1.94% (95% CI: 1.87–2.01). The breeds with the highest lipoma prevalence were Weimaraner (7.84%, 95% CI 6.46–9.40), Dobermann Pinscher (6.96%, 95% CI 5.67–8.44), German Pointer (5.23%, 95% CI 3.93–6.80), Springer Spaniel (5.19%, 95% CI 4.76–5.66), and Labrador Retriever (5.15%, 95% CI 4.90–5.41) (Fig. 1).

Of the lipoma cases with complete data available for that variable, 2004 (72.58%) were purebred, 1310 (47.40%) were female, 1756 (82.21%) were neutered and 661 (72.64%) were insured. Dogs with lipoma had a median adult bodyweight of 26.00 kg (IQR: 16.80–35.20) and median age was 10.02 years (IQR: 8.25–12.04). The most common breeds among the lipoma cases were Labrador Retriever (545, 19.71% of all confirmed cases), Springer Spaniel (182, 6.58%), Cocker Spaniel (130, 4.70%) and Staffordshire Bull Terrier (116, 4.20%), along with crossbred dogs (757, 27.38%) (Table 1).

**Table 1:** Descriptive and univariable logistic regression results for risk factors associated with lipoma diagnosis in dogs attending primary-care veterinary practices in the VetCompass™ Programme in the UK

Variable	Category	Case No. (%)	Control No. (%)	Odds ratio	95% CI^a^	Category *P*-value	Variable *P*-value
Purebred status	Crossbred	757 (0.81)	92,149 (99.19)	Base			< 0.001
	Purebred	2004 (0.70)	282,634 (99.30)	0.86	0.79–0.94	0.001	
Breed	Crossbred	757 (0.81)	92,149 (99.19)	Base			< 0.001
	Weimaraner	39 (3.06)	1237 (96.94)	3.84	2.77–5.32	< 0.001	
	Dobermann Pinscher	35 (2.63)	1294 (97.37)	3.29	2.34–4.64	< 0.001	
	German Pointer	19 (1.97)	944 (98.03)	2.45	1.55–3.88	< 0.001	
	Springer Spaniel	182 (1.96)	9118 (98.04)	2.43	2.06–2.86	< 0.001	
	Labrador Retriever	545 (1.95)	27,387 (98.05)	2.42	2.17–2.71	< 0.001	
	Cairn Terrier	22 (1.74)	1243 (98.26)	2.15	1.40–3.30	< 0.001	
	Border Collie	112 (1.09)	10,150 (98.91)	1.34	1.10–1.64	0.004	
	Golden Retriever	52 (1.08)	4746 (98.92)	1.33	1.00–1.77	0.046	
	Beagle	29 (1.02)	2802 (98.98)	1.26	0.87–1.83	0.225	
	Miniature Schnauzer	33 (1.00)	3261 (99.00)	1.23	0.87–1.75	0.243	
	Dalmatian	15 (1.00)	1481 (99.00)	1.23	0.74–2.06	0.424	
	Cocker Spaniel	130 (0.98)	13,099 (99.02)	1.21	1.00–1.46	0.048	
	Patterdale Terrier	19 (0.90)	2087 (99.10)	1.11	0.70–1.75	0.660	
	Rottweiler	33 (0.75)	4340 (99.25)	0.93	0.65–1.31	0.665	
	Border Terrier	29 (0.61)	4724 (99.39)	0.75	0.52–1.08	0.125	
	Boxer	30 (0.57)	5276 (99.43)	0.69	0.48–1.00	0.049	
	Other purebreds	307 (0.51)	60,029 (99.49)	0.62	0.55–0.71	< 0.001	
	Jack Russell Terrier	107 (0.47)	22,826 (99.53)	0.57	0.47–0.70	< 0.001	
	Staffordshire Bull Terrier	116 (0.44)	25,966 (99.56)	0.54	0.44–0.66	< 0.001	
	West Highland White Terrier	41 (0.40)	10,135 (99.60)	0.49	0.36–0.67	< 0.001	
	Breed not recorded	4 (0.37)	1064 (99.63)	0.46	0.17–1.22	0.120	
	Bichon	16 (0.30)	5402 (99.70)	0.36	0.22–0.59	< 0.001	
	Cavalier King Charles Spaniel	21 (0.24)	8684 (99.76)	0.29	0.19–0.45	< 0.001	
	German Shepherd Dog	20 (0.18)	10,799 (99.82)	0.23	0.14–0.35	< 0.001	
	Yorkshire Terrier	21 (0.16)	12,808 (99.84)	0.20	0.13–0.31	< 0.001	
	Lhasa Apso	9 (0.15)	5996 (99.85)	0.18	0.09–0.35	< 0.001	
	Shih Tzu	15 (0.12)	12,617 (99.88)	0.14	0.09–0.24	< 0.001	
	Chihuahua	7 (0.07)	9755 (99.93)	0.09	0.04–0.18	< 0.001	
	Pug	0 (0.00)	4428 (100.00)	~	~	~	
Kennel Club Breed Group	Breed not Kennel Club recognised	906 (0.73)	123,756 (99.27)	Base			< 0.001
	Toy	78 (0.16)	47,485 (99.84)	0.22	0.18–0.28	< 0.001	
	Utility	119 (0.31)	38,173 (99.69)	0.43	0.35–0.52	< 0.001	
	Terrier	259 (0.53)	48,432 (99.47)	0.73	0.64–0.84	< 0.001	
	Gundog	1016 (1.66)	60,116 (98.34)	2.31	2.11–2.53	< 0.001	
	Hound	86 (0.64)	13,315 (99.36)	0.88	0.71–1.10	0.268	
	Pastoral	173 (0.69)	25,034 (99.31)	0.94	0.80–1.11	0.489	
	Working	124 (0.67)	18,472 (99.33)	0.92	0.76–1.11	0.367	
Adult (> 18 months) bodyweight (kg)	< 10.0	200 (0.24)	83,327 (99.76)	Base			< 0.001
	10.0–19.9	666 (0.86)	77,090 (99.14)	3.60	3.07–4.22	< 0.001	
	20.0–29.9	721 (1.25)	56,792 (98.75)	5.29	4.52–6.19	< 0.001	
	30.0–39.9	697 (1.77)	38,609 (98.23)	7.52	6.42–8.81	< 0.001	
	≥ 40.0	364 (1.73)	20,657 (98.27)	7.34	6.17–8.73	< 0.001	
	Unrecorded	117 (0.12)	99,372 (99.88)	0.49	0.39–0.62	< 0.001	
Bodyweight relative to breed mean	Lower	862 (0.56)	154,194 (99.44)	Base			< 0.001
	Equal/Higher	1787 (1.44)	122,132 (98.56)	2.62	2.41–2.84	< 0.001	
	Unrecorded	116 (0.12)	99,952 (99.88)	0.21	0.17–0.25	< 0.001	
Age (years)	< 3.0 years	24 (0.02)	142,327 (99.98)	0.11	0.07–0.18	< 0.001	< 0.001
	3.0 - < 6.0 years	145 (0.15)	98,128 (99.85)	Base			
	6.0 - < 9.0 years	797 (1.23)	64,241 (98.77)	8.40	7.03–10.02	< 0.001	
	9.0 - < 12.0 years	1091 (2.75)	38,600 (97.25)	19.13	16.08–22.75	< 0.001	
	≥ 12.0 years	697 (2.48)	27,377 (97.52)	17.23	14.40–20.62	< 0.001	
	Unrecorded	11 (0.21)	5174 (99.79)	1.44	0.78–2.66	< 0.001	
Sex	Female	1310 (0.72)	181,347 (99.28)	Base			< 0.001
	Male	1454 (0.75)	103,177 (99.25)	1.04	0.97–1.12	0.282	
	Not recorded	1 (0.08)	1323 (99.92)	0.10	0.01–0.74	0.024	
Neuter	Entire	380 (0.27)	139,083 (99.73)	Base			< 0.001
	Neutered	1756 (1.04)	166,332 (98.96)	3.86	3.46–4.32	< 0.001	
	Unrecorded	629 (0.89)	70,432 (99.11)	3.27	2.88–3.71	< 0.001	
Sex-Neuter	Female-entire	172 (0.26)	65,611 (99.74)	Base			< 0.001
	Female-neutered	846 (1.01)	82,506 (98.99)	3.91	3.32–4.61	< 0.001	
	Female-unrecorded	292 (0.87)	33,230 (99.13)	3.35	2.78–4.05	< 0.001	
	Male-entire	208 (0.29)	72,573 (99.71)	1.09	0.89–1.34	0.387	
	Male-neutered	910 (1.08)	83,707 (98.92)	4.15	3.52–4.88	< 0.001	
	Male-unrecorded	336 (0.90)	36,897 (99.10)	3.47	2.89–4.18	< 0.001	
	Unrecorded-unrecorded	1 (0.08)	1323 (99.92)	0.29	0.04–2.06	0.215	
Insurance	Non-insured	249 (0.97)	25,364 (99.03)	Base			< 0.001
	Insured	661 (1.50)	43,293 (98.50)	1.56	1.34–1.80	< 0.001	
	Unrecorded	1855 (0.60)	307,109 (99.40)	0.62	0.54–0.70	< 0.001	

Row percentages shown in brackets. ^a^CI confidence interval

Of the non-case dogs with complete data on the variable, 282,634 (75.41%) were purebred, 181,347 (48.42%) were female, 166,332 (54.46%) were neutered and 43,293 (63.06%) were insured. The median adult bodyweight for non-cases was 16.50 kg (IQR: 9.00–28.00) and the median age was 4.18 years (IQR: 1.80–7.59). The most common breeds among the non-case dogs were Labrador Retriever (27,387, 7.29% of all non-cases), Staffordshire Bull Terrier (25,966, 6.91%), Jack Russell Terrier (22,826, 6.07) and Cocker Spaniel (13,099, 3.49%) accompanied by a substantial population of crossbred dogs (92,149, 24.52%) (Table 1). Data completeness varied between the variables assessed: breed 99.7%, age 98.64%, sex 99.7%, bodyweight at any age 88.96%, insurance 18.54%, and neuter 81.16%.

All tested variables were liberally associated with lipoma in univariable logistic regression modelling and were further evaluated in the main multivariable logistic regression modelling. The final main breed multivariable model retained five risk factors: *breed, bodyweight relative to breed mean*, *age, sex-neuter* and *insurance* (Table 2). An interaction effect was noted between sex and neuter status so these variables were combined into a single variable that reported the various sex-neuter permutations results separately. No other biologically significant interactions remained in the final model. The final model showed good fit and discrimination (area under the ROC curve: 0.8905, Hosmer-Lemeshow test: *P* = 0.631).

**Table 2:** Final breed multivariable logistic regression model for risk factors associated with diagnosis of lipoma in dogs attending primary-care veterinary practices in the VetCompass™ Programme in the UK

Variable	Category	Odds ratio	95% CI^a^	*P*-value
Breed	Crossbreed	1.00	–	
	Dobermann Pinscher	3.55	2.49–5.06	< 0.001
	Weimaraner	3.16	2.26–4.42	< 0.001
	Labrador Retriever	2.19	1.96–2.46	< 0.001
	Springer Spaniel	2.15	1.82–2.54	< 0.001
	Beagle	2.03	1.39–2.97	< 0.001
	German Pointer	2.03	1.27–3.25	0.003
	Miniature Schnauzer	1.52	1.07–2.18	0.021
	Cairn Terrier	1.44	0.93–2.22	0.100
	Cocker Spaniel	1.26	1.04–1.53	0.016
	Patterdale Terrier	1.25	0.79–1.99	0.340
	Rottweiler	1.09	0.76–1.55	0.636
	Border Collie	1.04	0.85–1.27	0.715
	Pug	1.00	–	
	Dalmatian	0.95	0.56–1.59	0.842
	Golden Retriever	0.83	0.63–1.10	0.209
	Breed not recorded	0.72	0.27–1.97	0.525
	Other purebreds	0.70	0.61–0.80	0.000
	Border Terrier	0.70	0.48–1.02	0.060
	Boxer	0.61	0.42–0.89	0.010
	Staffordshire Bull Terrier	0.60	0.49–0.73	< 0.001
	Jack Russell Terrier	0.49	0.40–0.60	< 0.001
	Bichon	0.45	0.28–0.75	0.002
	West Highland White Terrier	0.30	0.22–0.41	< 0.001
	Cavalier King Charles Spaniel	0.29	0.18–0.44	< 0.001
	Chihuahua	0.26	0.13–0.56	< 0.001
	Shih-tzu	0.24	0.14–0.40	< 0.001
	German Shepherd Dog	0.21	0.14–0.33	< 0.001
	Lhasa Apso	0.20	0.11–0.39	< 0.001
	Yorkshire Terrier	0.17	0.11–0.26	< 0.001
Bodyweight relative to breed mean	Lower	1.00	–	
	Equal/Higher	1.97	1.81–2.14	< 0.001
	Unrecorded	0.53	0.43–0.64	< 0.001
Age (years)	< 3.0 years	0.18	0.12–0.28	< 0.001
	3.0 - < 6.0 years	1.00	–	
	6.0 - < 9.0 years	7.56	6.33–9.04	< 0.001
	9.0 - < 12.0 years	17.52	14.71–20.85	< 0.001
	> or = 12.0 years	18.34	15.30–21.98	< 0.001
	No age available	3.45	1.84–6.45	< 0.001
Sex-Neuter	Female-Entire	1.00	–	
	Female-Neutered	1.62	1.37–1.91	< 0.001
	Female-Unknown	1.41	1.16–1.72	0.001
	Male-Entire	0.79	0.65–0.97	0.025
	Male-Neutered	1.99	1.69–2.36	< 0.001
	Male-Unknown	1.43	1.18–1.74	< 0.001
	Unknown-Unknown	0.82	0.11–6.04	0.844
Insurance	Uninsured	1.00	–	
	Insured	1.78	1.53–2.07	< 0.001
	Unknown	1.18	1.02–1.36	0.027

^a^CI confidence interval

After accounting for the effects of the other variables evaluated, 8 breeds showed increased odds of lipoma compared with crossbred dogs. The breeds with the highest odds included the Dobermann Pinscher (OR: 3.55, 95% CI 2.49–5.06, *P* <  0.001), Weimaraner (OR: 3.16, 95% CI 2.26–4.42, *P* <  0.001), Labrador Retriever (OR: 2.19, 95% CI 1.96–2.46, *P* <  0.001) and Springer Spaniel (OR: 2.15, 95% CI 1.82–2.54, *P* <  0.001). There were 11 breeds with reduced odds of lipoma compared with crossbreds. Individual dogs with an adult bodyweight that was equal or higher than their breed/sex mean had 1.96 (95% CI 1.81–2.14, *P* <  0.001) times the odds of lipoma compared with dogs that weighed below their breed/sex mean. Advancing age was strongly associated with increasing odds of lipoma. Compared with dogs aged 3.0 to < 6.0 years, dogs aged 9.0 - < 12.0 years had 17.52 times the odds (95% CI 14.71–20.85, *P* <  0.001) of lipoma. Neutered males (OR: 1.99, 95% CI 1.69–2.36, *P* <  0.001) and neutered females (OR: 1.62, 95% CI 1.37–1.91, *P* <  0.001) had higher odds than entire females. Insured dogs had 1.78 (95% CI 1.53–2.07, *P* <  0.001) times the odds of lipoma compared with uninsured dogs (Table 2).

As described in the methods, 3 variables (*purebred, Kennel Club breed group* and *adult bodyweight*) individually replaced the *breed* variable in the final multivariable model. Purebred dogs had 1.16 times the odds (95% CI 1.07–1.26, *P* = 0.001) compared with crossbred dogs. Of the seven Kennel Club breed groups, only Gundogs (OR: 2.08, 95% CI 1.90–2.28, *P* <  0.001) showed higher odds of lipoma compared with dogs of breeds that are not recognized by the Kennel Club, while the Toy, Utility, Terrier and Pastoral groups all had reduced odds. The odds of lipoma increased as adult bodyweight increased (Table 3).

**Table 3:** Results for variables that individually replaced the breed variable in the final multivariable logistic regression model (with age, bodyweight relative to breed mean, sex-neuter and insurance status) to evaluate risk factors associated with a diagnosis of lipoma in dogs attending primary-care veterinary practices in the VetCompass™ Programme in the UK

Variable	Category	Odds ratio	95% CI^a^	Category *P*-value
Purebred status	Crossbred	Base		
	Purebred	1.16	1.07–1.26	0.001
Kennel Club Breed Group	Breed not KC-recognised	Base		
	Toy	0.28	0.22–0.36	< 0.001
	Utility	0.57	0.47–0.69	< 0.001
	Terrier	0.65	0.56–0.75	< 0.001
	Gundog	2.08	1.90–2.28	< 0.001
	Hound	0.86	0.69–1.08	0.187
	Pastoral	0.78	0.66–0.92	0.004
	Working	1.12	0.93–1.36	0.233
Adult (> 18 months) bodyweight (kg)	< 10.0	Base		
	10.0–19.9	2.85	2.43–3.34	< 0.001
	20.0–29.9	4.00	3.41–4.68	< 0.001
	30.0–39.9	5.62	4.79–6.59	< 0.001
	≥ 40.0	5.85	4.90–6.97	< 0.001
	Unrecorded	1.14	0.90–1.44	0.265

^a^CI confidence interval

## Discussion

This is the first study to explore the wider presentation of lipomas in dogs attending primary-care practices by analysing clinical data from a multi-centre primary-care research database. The previous veterinary literature on lipomas is dominated by isolated case reports on referral cases describing unusual clinical signs, such as coughing due to an intra-thoracic lipoma or abnormalities related to nerve root compression [5, 34]. Instead, the current study explored a large count of routinely diagnosed cases in primary-care practice by evaluating the medical records of 384,284 dogs and identifying 2765 lipoma cases from a random subset of 36.5% of these dogs. It therefore, provides novel insights into the prevalence and risk factors associated with lipomas in the general population of dogs in the UK. A deficiency of information on disease occurrence in the wider dog population has previously been identified as a critical impediment to applying an evidence-based approach to disease prioritisation and breed health reforms in dogs. Analyses of primary-care veterinary clinical data have been suggested as a reliable source of such information [35–38].

This study based on veterinary clinical records estimated a one-year period prevalence of 1.94% of lipoma across all dog types in the UK. This is lower than the results of an owner questionnaire study evaluating pedigree dogs in the UK registered with The Kennel Club (KC) that reported a 4.3% lipoma prevalence [9]. The questionnaire study asked owners to report on conditions that they believed the dog had at any time point and therefore reported a current-lifetime prevalence which would anticipate higher values than the current study which reported a one-year period prevalence. The questionnaire study also relied on owners recall of previous conditions and included conditions that the owners ‘diagnosed’ themselves without necessarily including veterinary input so the results may have been influenced by some misclassification and recall bias effects [32]. However, the results did single out lipoma as the most common owner-recalled disorder in pedigree dogs and suggests that owners are highly conscious of lipoma masses and retain recall for their occurrence over long periods. In consequence, however benign that lipomas may behave clinically, it is likely that owners find these masses as highly noteworthy and even alarming, and therefore veterinarians should be especially explicit in their explanations of the clinical significance of these masses to clients.

The current study investigated the general dog population under veterinary care, which included pedigreed and non-pedigreed purebreds as well as designer-types and unspecified crossbreed dogs. Our study reported a 1.16 times higher odds of lipoma in purebred dogs compared with crossbred dogs which may further explain the higher prevalence in the KC-registered pedigree dogs of the questionnaire study. It should be noted that the one-year period prevalence reported in the current paper described the probability of evidence in the clinical records that confirmed the presence of lipoma at any time during the one-year 2013 study period. This value includes both cases that were pre-existing to 2013 as well as cases that were newly diagnosed (i.e. incident) during 2013. Consequently, period prevalence values are generally higher than incident risks for the same period and these are therefore not directly comparable metrics [32]. It should also be noted that there may have been animals with lipoma in the underlying population that were not recorded with the condition in the clinical notes (e.g. the dog was not presented for veterinary care during 2013 or the diagnosis was missed by the attending veterinary surgeon) and therefore the true one-year period prevalence of lipoma may be higher than the value reported here.

The current study had a particular interest in breed risk factors for lipoma. At this point, it is worth noting an inferential distinction between breeds that comprise a high proportion of lipoma caseloads in a clinical setting (i.e. influenced by being both/or a proportionately common breed and a proportionately commonly-affected breed) and breeds that are predisposed to lipoma (influenced by being a proportionately commonly-affected breed). Common presentation in veterinary clinics with a specific condition can reflect varying relative contributions from the overall popularity of the breed, confounding factors (e.g. insurance status, age structure of the breed in the local underlying pet population) as well as innate breed predisposition. For example, Labrador Retrievers are the most common dog breed in the UK and therefore would be expected to comprise a greater proportion of clinical caseloads than a less common breed such as the Weimaraner for disorders where these two breeds shared the same level of innate predisposition [10]. This distinction underlies the cognitive bias, which could be called a “presentation bias”, whereby common breeds are often perceived as predisposed just because they are commonly observed with certain disorders in clinical practice [39]. The current study shows a nice example of this presentation bias effect; the Labrador Retriever was the most commonly presented breed (19.71% of all cases) compared with the Weimaraner that had fewer than one-tenth of this case count (1.41% of all cases). However, the Weimaraner had a higher within-breed prevalence (7.84%) than the Labrador Retriever (5.15%) (Fig. 1). After taking other associated confounding factors into account such as relative bodyweight, age, sex-neuter and insurance, the Weimaraner still had higher odds of lipoma than the Labrador Retriever (OR: 3.16 vs. 2.19) (Table 2). These results emphasise the value of a thorough epidemiological analysis to get a truer understanding of breed health and highlight the risks of cognitive biases in personal perception [40].

A genetic hereditary component to developing lipomas overall has previously been suggested [41]. Certain dog breeds such as Labrador Retriever and Dobermann Pincher are regularly cited with higher risk of lipoma but following the literature back to the original source studies identifies that these claims are based on very weak evidence [42–44]. In contrast, the current study provides strong evidence of breed predilections for lipoma. After accounting for other confounding factors, there were 8 breeds that showed predisposition compared with crossbreds: Dobermann Pinscher, Weimaraner, Labrador Retriever, Springer Spaniel, Beagle, German Pointer, Miniature Schnauzer and Cocker Spaniel. It is also noteworthy that 5 of the 8 predisposed breeds are classified in the Kennel Club Gundog Group: Weimaraner, Labrador Retriever, Springer Spaniel, German Pointer, and Cocker Spaniel. Indeed, the Gundog Group was the only Kennel Club group with increased odds of lipoma, showing 2.08 times the odds compared with dogs that were not breed-recognised by the Kennel Club. The Gundog Group is divided into four categories (Retrievers; Spaniels; Hunt/Point/Retrieve; Pointers/Setters) and includes dogs that were originally trained to find live game and/or to retrieve game that had been shot and wounded [45]. Such breeds may have been selected to work in wintry adverse weather conditions, spending extended periods stationary to avoid scaring the game whilst also retaining high athletic ability on sudden request. It is possible that these dual working demands selected for specific adipose characteristics; for example, with differing propositions of isoforms of adipose uncoupling proteins or ratio of brown to white fat [46–48]. The Kennel Club describes the Gundog Group as good companion animals with temperaments ideal as all-round family dogs, suggesting that the majority of current generations of Gundogs are non-working but are instead owned as family pets [45]. It may be that the original adipose selection processes as working animals combined with the more sedentary and highly nourished life of the modern pet dog combine to expose an increased tendency to lipoma. It is also noteworthy that many of the predisposed breeds share a similar body conformation: medium–to-large bodysize, barrel chest and tapered abdomen and a smooth hair coat [30]. These features may facilitate identification of subcutaneous masses meaning that lipomas at these locations are easier to recognise in these breeds and therefore contribute to recognition bias in these breeds.

To date in the veterinary literature, the majority of breed-focussed disease studies have reported only positive predisposition to disease. This approach supports the identification of breeds with increased risk of disease that may undergo breed health reforms to try to breed away from some risk attributes [35]. It is also worth considering setting an alternative research goal that instead identifies breeds that are negatively predisposed to disease (i.e. protected). Greater understanding of why certain breeds or dog types do *not* get disease may offer as much, if not more, welfare progress than tunnel-vision focus on the predilected breeds [15]. The current study embraced this second approach and identified 11 breeds with lower lipoma odds than crossbreds: Yorkshire Terrier, Lhasa Apso, German Shepherd Dog, Shih-tzu, Chihuahua, Cavalier King Charles Spaniel, West Highland White Terrier, Bichon, Jack Russell Terrier, Staffordshire Bull Terrier and Boxer. These protected breeds do not include a single Gundog Group breed and have noticeably different body conformation to the predisposed breeds, tending to be smaller in bodysize and to have less pronounced proportional difference between the thorax and abdomen. Further research the genetics of adipose, and differential fat function and accumulation across the predisposed and protected dog breeds identified in the current study is warranted and may lead to substantial new discovery of lipoma pathogenetic pathways.

The current study explored associations between bodysize and lipoma. Body condition score data were not available so no conclusive inference can be drawn from these results on associations between obesity and lipoma. However, data were available that characterised the adult bodyweight of individual dogs as either below or equal/above the mean adult bodyweight for their breed and sex within the overall study population. This variable allowed the effect of low versus high adult bodyweight to be assessed after taking into account breed and sex. Among other reasons, a high bodyweight could reflect enhanced muscular mass, a large body frame or overweight/obesity. Dogs weighing at or above the mean for their breed and sex had 1.97 times the odds of diagnosis with lipoma. This supports the study hypothesis and suggests value in future exploration of lipoma association with obesity/overweight since the latter is a modifiable risk factor. This approach is also supported by published evidence of predisposition to obesity in some of the breeds that were also identified with high odds of lipoma in the current study including Cocker Spaniel [49] and Labrador Retrievers [50].

This study also identified a substantial and strong trend towards increasing odds of lipoma as adult bodyweight increased. This may reflect a true increase in odds of cellular metaplasia or neoplasia with increasing bodyweight. Osteosarcoma in dogs has similarly been linked to increasing bodyweight, although the biological mechanisms may be different for the different neoplasms [41].

Advancing age has previously been identified as a risk factor for neoplasia in general [41, 51]. Specifically, adenocarcinoma/adenoma, melanocytic tumour and squamous cell carcinoma diagnosis increased with age in a large study using the Swiss Canine Cancer Registry [51] and osteosarcoma risk increased with age in Greyhounds in the US [52]. It is possible that the same is true for risk of lipoma development. The current study reports the median age of lipomas cases was 10.02 years compared with the median age of 4.18 years for non-lipoma dogs. The odds of lipoma also increased markedly as dogs aged, with dogs aged 9–12 years having 17.52 times the odds compared with dogs aged less than 3 years. There is a strong case to be made that lipoma should be included as one of the accepted common diseases of aging in dogs [53]. Risk factors for lipoma development in people are reported to be similar to the findings of our study in dogs although there is also a paucity of literature on the occurrence of lipomas in people. In humans, the incidence of lipomas is increased in patients with obesity, hyperlipidemia, and diabetes mellitus [54]. A genetic component is suspected for lipoma development in people [55]. A genetic link has not been reported in dogs but the breed predisposition found in this study suggests that a genetic component to the risk of developing lipomas is also likely to exist in dogs.

The influences of sex hormones on tumour development is complex; neuter status has been reported with differing effects on different tumour types and to influence the risk of developing both genital and non-genital neoplasia [56]. For example non-ovariectomised bitches have been reported at increased risk of developing mammary carcinoma and castrated male dogs at increased risk of prostatic carcinoma [12, 13, 56–60]. However, Rottweilers undergoing early gonadectomy (before 12 months of age) were reported at increased risk of osteosarcoma [56, 61, 62]. There is little prior evidence on the effects of sex and neuter status on the risk of lipoma. The current study identified reduced risk of lipoma in entire females and entire males compared with neutered females and neutered males, even after taking age into account. This could indicate some protective effects of female and male sex hormones. However, post-neutering changes in fat distribution and decreased energy requirements have been demonstrated and the effects of neutering on lipoma risk may be mediated by obesity as a confounder rather than directly [63].

Insured dogs had 1.78 times greater odds of lipoma diagnosis compared with uninsured dogs. This association is likely to reflect increased diagnostic recognition mediated by owner and financial factors rather than any intrinsic increased disease risk in insured dogs. Relaxation on financial constraints to presentation for veterinary care, diagnostic procedures and surgical management through insurance has similarly been shown to increase diagnostic probability in many other conditions [64–69].

### Limitations

This study was limited by its retrospective nature and the use of externally recorded clinical data; which may have led to some disease status misclassification. This study may have underrepresented lipoma because true cases that were not presented for veterinary care during 2013 were not included as cases. Alternatively, lipoma could be over-represented because the study did not require laboratory confirmation of lipoma cases; although the characteristic presenting phenotype of lipoma cases suggests that diagnosis based on clinical examination alone is likely to have a high positive predictive value [70]. This study excluded dogs that were not under veterinary care and therefore may have introduced bias toward the increasingly neutered, insured and more closely monitored subset of the population under veterinary care. As discussed above, body condition scores were not available for this study and therefore analysis of association between obesity and lipoma was not possible. The analyses did not take account of effects from differing counts or location of lipoma masses across the population and it is possible that these features also vary across breeds and other demographic risk factors.

## Conclusions

This large population study is the first study to focus primarily on lipoma occurrence in the dogs in the UK. Lipoma has been confirmed as a common clinical occurrence with a one-year prevalence of 1.94%. Strong breed associations for both lipoma predisposition and protection were identified that can assist with breed health reforms as well as contributing to the basic scientific understanding of lipoma development. Heavier, older, neutered and insured dogs also had higher odds of diagnosis. Lipoma detection should be included as a routine part of veterinary clinical exanimation, especially in breeds identified as high-risk here.

## Abbreviations

CI: Confidence interval
EPR: Electronic patient record
FNA: Fine needle aspiration
IQR: Interquartile range
KC: The Kennel Club
OR: Odds ratio

## References

[1] Van Nimwegen S, Kirpensteijn J, Tobias KM, Johnston SA (2017). Specific disorders. Veterinary surgery : small animal.

[2] Spoldi E, Schwarz T, Sabattini S, Vignoli M, Cancedda S, Rossi F (2017). Comparisons among computed tomographic features of adipose masses in dogs and cats. Vet Radiol Ultrasound.

[3] Meuten DJ, Meuten DJ (2002). Tumors in domestic animals.

[4] Avallone G, Roccabianca P, Crippa L, Lepri E, Brunetti B, Bernardini C (2016). Histological classification and Immunohistochemical evaluation of MDM2 and CDK4 expression in canine Liposarcoma. Vet Pathol.

[5] Lynch S, Halfacree Z, Desmas I, Cahalan SD, Keenihan EK, Lamb CR (2013). Pulmonary lipoma in a dog. J Small Anim Pract.

[6] Kraun MB, Nelson NC, Hollinger C (2015). Imaging diagnosis—computed tomographic, surgical, and histopathologic characteristics of an infiltrative angiolipoma in a dog. Vet Radiol Ultrasound.

[7] Liptak J, Forrest L, Withrow S, Vail D, Page R (2013). Soft Tissue Sarcomas. Small Animal Clinical Oncology.

[8] Thomson MJ, Withrow SJ, Dernell WS, Powers BE (1999). Intermuscular lipomas of the thigh region in dogs: 11 cases. J Am Anim Hosp Assoc.

[9] Wiles BM, Llewellyn-Zaidi AM, Evans KM, O'Neill DG, Lewis TW (2017). Large-scale survey to estimate the prevalence of disorders for 192 kennel Club registered breeds. Canine Genet Epidemiol.

[10] O'Neill DG, Church DB, McGreevy PD, Thomson PC, Brodbelt DC (2014). Prevalence of disorders recorded in dogs attending primary-care veterinary practices in England. PLoS One.

[11] Boerkamp K, Teske E, Boon L, Grinwis G, van den Bossche L, Rutteman G (2014). Estimated incidence rate and distribution of tumours in 4,653 cases of archival submissions derived from the Dutch golden retriever population. BMC Vet Res.

[12] Brønden LB, Nielsen SS, Toft N, Kristensen AT (2010). Data from the Danish veterinary Cancer registry on the occurrence and distribution of neoplasms in dogs in Denmark. Vet Rec.

[13] Dobson JM, Samuel S, Milstein H, Rogers K, Wood JLN (2002). Canine neoplasia in the UK: estimates of incidence rates from a population of insured dogs. J Small Anim Pract.

[14] Hendrick M, Meuten DJ (2017). Mesenchymal Tumours of the skin and soft tissues. Tumors in domestic animals.

[15] Gough A, Thomas A, O'Neill D (2018). Breed predispositions to disease in dogs and cats.

[16] O'Neill D, Church D, McGreevy P, Thomson P, Brodbelt D (2014). Approaches to canine health surveillance. Canine Genet Epidemiol.

[17] McGreevy PD, Nicholas FW (1999). Some practical solutions to welfare problems in dog breeding. Anim Welf.

[18] Harris Georgina L., Brodbelt David, Church David, Humm Karen, McGreevy Paul D., Thomson Peter C., O'Neill Dan (2018). Epidemiology, clinical management, and outcomes of dogs involved in road traffic accidents in the United Kingdom (2009-2014). Journal of Veterinary Emergency and Critical Care.

[19] Anderson KL, O'Neill DG, Brodbelt DC, Church DB, Meeson RL, Sargan D (2018). Prevalence, duration and risk factors for appendicular osteoarthritis in a UK dog population under primary veterinary care. Sci Rep.

[20] O'Neill D. G., O'Sullivan A. M., Manson E. A., Church D. B., Boag A. K., McGreevy P. D., Brodbelt D. C. (2017). Canine dystocia in 50 UK first-opinion emergency-care veterinary practices: prevalence and risk factors. Veterinary Record.

[21] O'Neill DG, Riddell A, Church DB, Owen L, Brodbelt DC, Hall JL (2017). Urinary incontinence in bitches under primary veterinary care in England: prevalence and risk factors. J Small Anim Pract.

[22] O'Neill DG, Lee MM, Brodbelt DC, Church DB, Sanchez RF (2017). Corneal ulcerative disease in dogs under primary veterinary care in England: epidemiology and clinical management. Canine Genet Epidemiol.

[23] The VeNom Coding Group: VeNom Veterinary Nomenclature [http://www.venomcoding.org]. Accessed 13 Sept 2018.

[24] O'Neill DG, Scudder C, Faire JM, Church DB, McGreevy PD, Thomson PC (2016). Epidemiology of hyperadrenocorticism among 210,824 dogs attending primary-care veterinary practices in the UK from 2009 to 2014. J Small Anim Pract.

[25] Pearce N (2012). Classification of epidemiological study designs. Int J Epidemiol.

[26] Epi Info 7 CDC: Centers for Disease Control and Prevention (US): Introducing Epi Info 7 [http://wwwn.cdc.gov/epiinfo/7]. Accessed 13 Sept 2018.

[27] Murray JK, Browne WJ, Roberts MA, Whitmarsh A, Gruffydd-Jones TJ (2010). Number and ownership profiles of cats and dogs in the UK. Vet Rec.

[28] Irion DN, Schaffer AL, Famula TR, Eggleston ML, Hughes SS, Pedersen NC (2003). Analysis of genetic variation in 28 dog breed populations with 100 microsatellite markers. J Hered.

[29] Scott M, Flaherty D, Currall J (2012). Statistics: how many?. J Small Anim Pract.

[30] The Kennel Club: Breed Information Centre [http://www.thekennelclub.org.uk/services/public/breed/]. Accessed 13 Sept 2018.

[31] Kirkwood BR, Sterne JAC (2003). Essential medical statistics.

[32] Dohoo I, Martin W, Stryhn H (2009). Veterinary epidemiologic research.

[33] Hosmer DW, Lemeshow S, Sturdivant RX (2013). Applied logistic regression.

[34] Wahle AM, Raith K, Posch B, Eddicks L, Matiasek K, Jurina K (2016). Imaging diagnosis - concentric periradicular lipoma causing lumbar nerve root compression in a dog. Vet Radiol Ultrasound.

[35] Bateson P (2010). Independent inquiry into dog breeding.

[36] Rooney NJ (2009). The welfare of pedigree dogs: cause for concern. J Vet Behav.

[37] McGreevy PD (2007). Breeding for quality of life. Anim Welf.

[38] Collins LM, Asher L, Summers JF, Diesel G, McGreevy PD (2010). Welfare epidemiology as a tool to assess the welfare impact of inherited defects on the pedigree dog population. Anim Welf.

[39] Pines JM (2008). Profiles in patient safety: confirmation Bias in emergency medicine. Acad Emerg Med.

[40] Croskerry P (2003). The importance of cognitive errors in diagnosis and strategies to minimize them. Acad Med.

[41] Dobson Jane M. (2013). Breed-Predispositions to Cancer in Pedigree Dogs. ISRN Veterinary Science.

[42] Kim H-J, Chang H-S, Choi C-B, Song Y-S, Kim S-M, Lee J-S (2005). Infiltrative lipoma in cervical bones in a dog. J Vet Med Sci.

[43] Hobert MK, Brauer C, Dziallas P, Gerhauser I, Algermissen D, Tipold A (2013). Infiltrative lipoma compressing the spinal cord in 2 large-breed dogs. Can Vet J.

[44] Bergman PJ, Withrow SJ, Straw RC, Powers BE (1994). Infiltrative lipoma in dogs: 16 cases (1981-1992). J Am Vet Med Assoc.

[45] The Kennel Club: Breed Standards Information: Dog Breeds & Groups [https://www.thekennelclub.org.uk/activities/dog-showing/breed-standards/]. Accessed 13 Sept 2018.

[46] Jimenez AG (2016). Physiological underpinnings in life-history trade-offs in man’s most popular selection experiment: the dog. J Comp Physiol B.

[47] McKenzie EC, Hinchcliff KW, Valberg SJ, Williamson KK, Payton ME, Davis MS (2008). Assessment of alterations in triglyceride and glycogen concentrations in muscle tissue of Alaskan sled dogs during repetitive prolonged exercise. Am J Vet Res.

[48] Kajimura S, Seale P, Tomaru T, Erdjument-Bromage H, Cooper MP, Ruas JL (2008). Regulation of the brown and white fat gene programs through a PRDM16/CtBP transcriptional complex. Genes Dev.

[49] Lund EM, Armstrong PJ, Kirk CA, Klausner JS (2006). Prevalence and risk factors for obesity in adult dogs from private US veterinary practices. Int J Appl Res Vet Med.

[50] Mankowska M, Krzeminska P, Graczyk M, Switonski M (2017). Confirmation that a deletion in the POMC gene is associated with body weight of Labrador retriever dogs. Res Vet Sci.

[51] Grüntzig K, Graf R, Boo G, Guscetti F, Hässig M, Axhausen KW (2016). Swiss canine Cancer registry 1955–2008: occurrence of the most common tumour diagnoses and influence of age, breed, body size, sex and neutering status on tumour development. J Comp Pathol.

[52] Rosenberger JA, Pablo NV, Crawford PC (2007). Prevalence of and intrinsic risk factors for appendicular osteosarcoma in dogs: 179 cases (1996-2005). J Am Vet Med Assoc.

[53] Creevy Kate E., Austad Steven N., Hoffman Jessica M., O’Neill Dan G., Promislow Daniel E.L. (2016). The Companion Dog as a Model for the Longevity Dividend. Cold Spring Harbor Perspectives in Medicine.

[54] Bird JE, Morse LJ, Feng L, Wang W-L, Lin PP, Moon BS, et al. Non-radiographic risk factors differentiating atypical Lipomatous tumors from lipomas. Front Oncol. 2016;6(197):1-6. 10.3389/fonc.2016.00197 PMC5031604 27713864

[55] Kolb L, Rosario-Collazo JA (2018). Lipoma.

[56] Smith AN (2014). The role of neutering in Cancer development. Vet Clin.

[57] Egenvall A, Bonnett BN, Öhagen P, Olson P, Hedhammar Å, von Euler H (2005). Incidence of and survival after mammary tumors in a population of over 80,000 insured female dogs in Sweden from 1995 to 2002. Prev Vet Med.

[58] Merlo DF, Rossi L, Pellegrino C, Ceppi M, Cardellino U, Capurro C (2008). Cancer incidence in pet dogs: findings of the animal tumor registry of Genoa, Italy. J Vet Intern Med.

[59] Teske E, Naan E.C, van Dijk E.M, Van Garderen E, Schalken J.A (2002). Canine prostate carcinoma: epidemiological evidence of an increased risk in castrated dogs. Molecular and Cellular Endocrinology.

[60] Bryan JN, Keeler MR, Henry CJ, Bryan ME, Hahn AW, Caldwell CW (2007). A population study of neutering status as a risk factor for canine prostate cancer. Prostate.

[61] Cooley DM, Beranek BC, Schlittler DL, Glickman NW, Glickman LT, Waters DJ (2002). Endogenous gonadal hormone exposure and bone sarcoma risk. Cancer Epidemiol Biomarkers Prev.

[62] de la Riva GT, Hart BL, Farver TB, Oberbauer AM, Messam LLM, Willits N (2013). Neutering dogs: effects on joint disorders and cancers in Golden retrievers. PLoS One.

[63] Jeusette I, Detilleux J, Cuvelier C, Istasse L, Diez M (2004). Ad libitum feeding following ovariectomy in female beagle dogs: effect on maintenance energy requirement and on blood metabolites. J Anim Physiol Anim Nutr (Berl).

[64] O'Neill D.G., Elliott J., Church D.B., McGreevy P.D., Thomson P.C., Brodbelt D.C. (2013). Chronic Kidney Disease in Dogs in UK Veterinary Practices: Prevalence, Risk Factors, and Survival. Journal of Veterinary Internal Medicine.

[65] O'Neill DG, Meeson RL, Sheridan A, Church DB, Brodbelt DC (2016). The epidemiology of patellar luxation in dogs attending primary-care veterinary practices in England. Canine Genet Epidemiol.

[66] Shoop Stephanie JW, Marlow Stephanie, Church David B, English Kate, McGreevy Paul D, Stell Anneliese J, Thomson Peter C, O’Neill Dan G, Brodbelt David C (2015). Prevalence and risk factors for mast cell tumours in dogs in England. Canine Genetics and Epidemiology.

[67] Mattin M, O'Neill D, Church D, McGreevy PD, Thomson PC, Brodbelt D (2014). An epidemiological study of diabetes mellitus in dogs attending first opinion practice in the UK. Vet Rec.

[68] Mattin MJ, Boswood A, Church DB, López-Alvarez J, McGreevy PD, O'Neill DG (2015). Prevalence of and risk factors for degenerative mitral valve disease in dogs attending primary-care veterinary practices in England. J Vet Intern Med.

[69] Taylor-Brown FE, Meeson RL, Brodbelt DC, Church DB, McGreevy PD, Thomson PC (2015). Epidemiology of cranial cruciate ligament disease diagnosis in dogs attending primary-care veterinary practices in England. Vet Surg.

[70] Bacon N, Dobson J, BDX L (2011). Soft tissue sarcomas. BSAVA manual of canine and feline oncology.

